# Intranasal Vaccination With Recombinant Antigen-FLIPr Fusion Protein Alone Induces Long-Lasting Systemic Antibody Responses and Broad T Cell Responses

**DOI:** 10.3389/fimmu.2021.751883

**Published:** 2021-10-11

**Authors:** Ming-Shu Hsieh, Chia-Wei Hsu, Ling-Ling Tu, Kit Man Chai, Li-Lu Yu, Chiao-Chieh Wu, Mei-Yu Chen, Chen-Yi Chiang, Shih-Jen Liu, Ching-Len Liao, Hsin-Wei Chen

**Affiliations:** ^1^ National Institute of Infectious Diseases and Vaccinology, National Health Research Institutes, Miaoli, Taiwan; ^2^ Graduate Institute of Biomedical Sciences, China Medical University, Taichung, Taiwan; ^3^ Graduate Institute of Medicine, Kaohsiung Medical University, Kaohsiung, Taiwan

**Keywords:** intranasal vaccination, mucosal vaccines, Zika vaccines, Fcγ receptor, formyl peptide receptor-like 1 inhibitory protein (FLIPr)

## Abstract

A simple formulation is urgently needed for mucosal vaccine development. We employed formyl peptide receptor-like 1 inhibitory protein (FLIPr), an FcγR antagonist secreted by *Staphylococcus aureus*, as a vector to target ovalbumin (OVA) to dendritic cells (DCs) *via* intranasal administration. Our results demonstrate that intranasal administration of recombinant OVA-FLIPr fusion protein (rOVA-FLIPr) alone efficiently delivers OVA to DCs in nasal lymphoid tissue. Subsequently, OVA-specific IgG and IgA antibodies in the circulatory system and IgA antibodies in mucosal tissue were detected. Importantly, activation of OVA-specific CD4^+^ and CD8^+^ T cells and induction of a broad-spectrum cytokine secretion profile were detected after intranasal administration of rOVA-FLIPr alone in immunocompetent C57BL/6 mice. Furthermore, we employed immunodeficient AG129 mice as a Zika virus infection model and demonstrated that intranasal administration of recombinant Zika virus envelope protein domain III-FLIPr fusion protein induced protective immune responses against the Zika virus. These results suggest that antigen-FLIPr fusion protein alone *via* intranasal administration can be applied to mucosal vaccine development.

## Introduction

Several infections start on mucosal surfaces. For example, infection of influenza virus, severe acute respiratory syndrome coronavirus, Vibrio cholerae, and herpes simplex virus. Commonly vaccinated routes, including subcutaneous, intradermal, or intramuscular injection, can induce protective systemic antibodies but exhibit weaker responses in the induction of substantial mucosal antibodies (1). It is believed that mucosal immunity plays an important role in the first line of defense against pathogen invasion. However, only limited mucosal vaccines are currently available for human use. The development of effective mucosal vaccines that protect mucosal sites is needed (1–4).

The primary route of Zika virus transmission is well known to occur through the bite of infected Aedes species mosquitoes (5, 6), primarily Aedes aegypti and Aedes albopictus. Recently, several reports have indicated that Zika virus can also be transmitted through sexual contact (7–9). Many clinical studies have shown the presence of Zika virus RNA in the semen, vagina and cervix as well as vaginal and endocervical mucosa of infected patients (5, 9, 10). These results indicate that the induction of mucosal immunity is also important for novel Zika vaccine design.

Adoptive transfer of purified IgG from immunized mice provided passive protection, and depletion of CD4 and CD8 T cells in immunized mice did not abolish this protection (11). These results suggest that antibodies alone were able to protect Zika virus infection. However, T cells are an important arm for the host to defend viral infections. Several studies demonstrate that CD4 and CD8 T cells play a critical role to mediate protection against Zika virus infection (12–14). Therefore, an ideal Zika vaccine should induce robust antibody and T cell responses.

The majority of mucosal vaccines approved for humans are live-attenuated vaccines that are administered orally (2, 15). Live or live-attenuated vaccines are undesirable in immunocompromised infants, immunodeficient individuals and elderly people due to the risk of reversion to a virulent pathogen (16–19). Subunit vaccines using recombinant protein are noninfectious. They have significant safety advantages over live-attenuated vaccines; however, recombinant protein has weak immunogenicity. Extra adjuvant formulation is necessary for inducing robust immune responses. Novel mucosal adjuvants or a combination of traditional adjuvants may increase vaccine immunogenicity (20–22). Many adjuvants have been found to boost mucosal immune responses to antigens. A major category of mucosal adjuvants is Toll-like receptor (TLR) agonists. For example, CpG acts through TLR-9, which has been used in influenza A virus vaccines (23–26). Monophosphoryl lipid A and flagellin stimulate signals through TLR-4 and TLR-5, respectively (27–29). They have been used as adjuvants in mucosal vaccines. The second category of mucosal adjuvants is toxins, such as bacterial outer membrane protein (30), cholera toxin (31), or heat-labile enterotoxin (32). The use of bacterial toxins for intranasal administration has revealed some side effects, including Bell’s palsy and other adverse events related to facial nerve disease (33–35). Heat-labile enterotoxin is no longer recommended for use as a vaccine adjuvant. Therefore, selecting a suitable adjuvant able to satisfy both the safety and efficacy of mucosal vaccines is crucial.

Although new technologies or novel adjuvants for mucosal vaccines are being developed with the aim of enhancing local and systemic immune responses, delivery of antigen through the mucosal route is still a marked challenge at present. The major obstacles are not only associated with poor immunogenicity but also inefficient uptake and presentation by antigen-presenting cells, enzymatic degradation and risk of tolerance rather than protective immunity (36). Therefore, development of an appropriate approach is crucial to achieve effective mucosal immunization.

Dendritic cells (DCs) are professional antigen-presenting cells that are present throughout the body, including at mucosal surfaces. They play a critical role in linking the innate immune system with the adaptive immune system (36–38). Thus, targeting mucosal DCs is an effective strategy to induce mucosal immunity. FLIPr is an FcγR binding protein secreted by *Staphylococcus aureus* (39). We previously demonstrated that FLIPr is an effective carrier for targeting antigens to FcγRs. Immunization of antigen-FLIPr fusion protein *via* a subcutaneous route induces robust antigen-specific immune responses to suppress tumor growth (40, 41). Our results demonstrate that FLIPr is an efficacious vector for delivering antigens to DCs for the induction of potent immunity *via* subcutaneous administration without extra adjuvant formulation.

Targeting antigens to FcγR has been shown to trigger both systemic immune responses and mucosal immune responses after intranasal immunization (42–47). Although the merits of antigen-FlIPr fusion proteins have been demonstrated *via* subcutaneous administration, the capability of antigen-FlIPr fusion proteins to induce mucosal immunity *via* intranasal administration has not yet been tested. In this study, ovalbumin (OVA) was used as a model antigen to evaluate the capability of recombinant OVA-FlIPr fusion protein (rOVA-FLIPr) to elicit mucosal immunity and systemic immune responses *via* intranasal administration. Furthermore, the Zika virus infection model was used to investigate whether intranasal administration of recombinant Zika virus envelope protein domain III-FLIPr fusion protein (rZEIII-FLIPr) generated protective immune responses against the Zika virus. To the best of our knowledge, this is the first proof-of-concept study demonstrating that antigen-FLIPr fusion protein alone *via* intranasal administration achieves enhanced immunogenicity and improves protection against infection.

## Methods

### Construction of Expression Vector

Construction of the pOVA, pOVA-FLIPr and pZEIII expression vectors has been described previously (40, 48). The DNA sequence of ZEIII-FLIPr was synthesized (Purigo Biotechnology Co., Taipei, Taiwan) using *E. coli* codon usage according to the amino acid sequences of Zika virus PRVABC59 (accession number AMC13911) and FLIPr (accession number BAB57318). To construct the plasmid pZEIII-FLIPr, a forward primer, 5’-ACTGCGCATATGaaaggcgtgagc-3’ (the NdeI site is underlined), and a reverse primer, 5’ CACGAGCTCGAGatcccaataaatgctatc-3’ (the XhoI site is underlined), were used to clone the ZEIII-FLIPr sequence into the NdeI and XhoI sites of the plasmid pET-22b(+) (Novagen, Madison, WI). As a result, the C-terminus of rZEIII-FLIPr contained a hexahistidine tag (His-tag).

### Production of Recombinant Protein

The production and purification of rOVA, rOVA-FLIPr, and rZEIII was described previously (40, 48). For the expression of rZEIII-FLIPr, pZEIII-FLIPr was transformed into *E. coli* BL21 (DE3) (Lucigen, Middleton, WI). After transformation, the *E. coli* were cultured in Luria-Bertani broth at 37°C overnight. To scale up protein production, 10 ml of the overnight culture was added to 500 ml of medium in a 2-L shaker flask and incubated at 37°C until the OD600 of cultures reached 0.6. IPTG (1 mM) was added to induce protein expression, followed by incubation at 20°C for 20 hours. Cells were harvested and then disrupted in a French press (Constant Systems, Daventry, UK) at 27 Kpsi in homogenization buffer [20 mM Tris (pH 8.0), 50 mM sucrose, 500 mM NaCl and 10% glycerol]. The cell lysate was clarified by centrifugation (80,000×g for 40 min) as previously described (48). The majority of rZEIII-FLIPr was present in the inclusion bodies. rZEIII-FLIPr was extracted using extraction buffer [0.02 M Tris (pH 8.0), 0.05 M sucrose, 0.5 M NaCl, 10% glycerol and 3 M GuHCl]. The extracted fraction was loaded onto immobilized metal affinity chromatography (IMAC) columns (BIO-RAD, Hercules, CA) (2.5 cm i.d. × 10.0 cm) containing 20 ml Ni-NTA resin (Qiagen, San Diego, CA, USA) to bind rZEIII-FLIPr. The column was washed with a 15-fold column volume of extraction buffer containing 20 mM imidazole and then washed with a 150-fold column volume of 10 mM Na_2_HPO_4_ and 0.4 M NaCl containing 0.1% Triton X-114 to remove the lipopolysaccharide. Next, the column was washed with a 20-fold column volume of 10 mM Na_2_HPO_4_ to remove the residual Triton X-114, and then rZEIII-FLIPr was eluted with 10 mM Na_2_HPO_4_ containing 500 mM imidazole. The eluted rZEIII-FLIPr was dialyzed to 10 mM Na_2_HPO_4_ three times for at least 6 hours each time. The endotoxin levels of the purified rZEIII-FLIPr were determined using the Limulus amebocyte lysate assay (Associates of Cape Cod, Inc., Cape Cod, MA), and the resulting endotoxin levels were <7 EU/mg. After dialysis, the rZEIII-FLIPr was lyophilized and stored at -20°C. The fractions from each step were analyzed using SDS-PAGE and immunoblotted with anti-His tag antibodies.

### Mice

C57BL/6 mice were obtained from the National Laboratory Animal Breeding and Research Center (Taipei, Taiwan). AG129 mice, immunocompromised mice lacking the receptor for types I and II IFN (IFN α/β/γ), were bred at the Laboratory Animal Center of the National Health Research Institutes (NHRI). All mice were housed at the Laboratory Animal Center of the NHRI. All animal studies were approved and were performed in compliance with the guidelines of the Animal Committee of the NHRI.

### Immunohistochemistry and Immunofluorescence Staining of Nasal Cavity Tissue

For the preparation of nasal cavity samples, C57BL/6 mice were euthanized, and then with their heads immobilized, the lower jaw was removed together with the tongue. The snout was removed with a transverse cut behind the back molars by using a scalpel. After removing the skin and any excess soft tissue, the tissue was fixed in 4% paraformaldehyde for 2 hours at room temperature and then decalcified in 10% EDTA for approximately 7 days. The decalcified heads were immersed in 30% sucrose solution overnight at 4°C and then embedded in OCT (Sakura Finetek, Tokyo, Japan), quickly frozen on dry ice and stored at -80°C. The tissues were cut on a cryostat (Leica CM1950) (Leica Biosystems Nussloch GmbH, Heidelberger, Germany) into 12 μm slices. For  immunohistochemical staining, the nasal cryostat sections were then stained with hematoxylin and eosin. For immunofluorescence staining, nasal cryostat sections were fixed in paraformaldehyde for 20 min at room temperature followed by permeabilization with 0.1% Triton X-100 for 15 min. After 3 washes with 0.01% Tween/PBS for 5 min, the sections were blocked with 2.5% BSA in PBS for 30 min in a humidified chamber at room temperature. The antigen in the nasal cavity was detected by staining with mouse anti-His tag antibody (J099B12) (Biolegend, San Diego, CA) and then labeling with Alexa Fluor 488-conjugated goat anti-mouse Fc antibody (Poly4053) (Biolegend, San Diego, CA). Hoechst 33342 was used to stain the nucleus (Invitrogen, Waltham, MA).

### Isolation of NALT and NALT Single-Cell Preparation

The C57BL/6 mice were euthanized by inhalation of CO_2_. The angle of the mouth on both sides was incised using the established protocol to open the mouth and expose the upper palate (49). A surgical blade was used to carefully incise and excise the upper palate. The palate was gently and slowly peeled back using curved fine forceps and placed into individual wells in the first column of a sterile 24-well plate prefilled with 1 mL of 4°C complete culture medium (RPMI 1640 supplemented with 10% fetal bovine serum, 100 μg/mL streptomycin, 100 UI/mL penicillin, and 10 mM HEPES pH 7.4). To wash the palates, the forceps were used to move palates into each successive well in a row.

For separate analyses of NALT DCs, NALT was mechanically disrupted and transferred to conical tubes. The tissue pieces were resuspended in 1 mL of RPMI containing 0.4 mg/mL collagenase (Sigma-Aldrich, St. Louis, MO) and incubated for 30 min at 37°C. The tissue was ground, and the suspension was collected and passed through a 70 mm cell strainer. The cells were pelleted by centrifugation at 300×g for 5 min.

### Analysis of Antigen Uptake by NALT Dendritic Cells

rOVA and rOVA-FLIPr were labeled with an Alexa Fluor 647 labeling kit (Thermo Fisher Scientific, MA). Groups of C57BL/6 mice (6–8 weeks of age) were intranasally administered 30 μg Alexa647-labeled rOVA or rOVA-FLIPr. Two hours after administration, a single NALT cell suspension (pooled from 3 mice) was dissected out as mentioned in the preceding sections. The LIVE/DEAD fixable dead cell stain kit (Thermo Fisher) was used to evaluate the viability of NALT cells by flow cytometry. Lymphocytes were distinguished by staining with PE-Cy7-conjugated anti-CD45 antibody (30-F11). Dendritic cells were distinguished by staining with PerCP-Cy5.5-conjugated anti-MHCII antibody (M5/114.15.2) and PE-conjugated anti-CD11c antibody (N418). Staining antibodies were obtained from Biolegend. Antigen-positive DCs in draining NALT were analyzed by flow cytometry.

### Mouse Immunization and Sample Collection

Groups of C57BL/6 mice or AG129 mice (immunocompromised mice lacking the receptor for types I and II IFN, IFN α/β/γ) (6–8 weeks of age) were immunized with 30 μg/dose of each vaccine candidate *via* intranasal administration. Mice immunized with PBS were used as controls. All animals were immunized 3 times at 2-week intervals using the same regimen. Serum and vaginal lavage were collected from each mouse at different time points as indicated. BALF samples were collected 6 weeks after the first vaccination.

### Measurement of Antibody Titers

Antigen-specific IgG and IgA titers in the indicated samples were determined by titration as previously described with some modifications (48). Serum samples were prepared in 3-fold serial dilutions (starting at the indicated dilution) and then added to antigen-coated 96-well plates. Bound IgG and IgA were identified by a goat horseradish peroxidase-conjugated goat anti-mouse IgG Fc antibody (MP Biomedicals, Irvine, CA) and a horseradish peroxidase-conjugated goat anti-mouse IgA Fc antibody (Invitrogen), respectively. After washing with PBS and the addition of a 3,3’,5,5’-tetramethylbenzidine substrate, the absorbance at 450 nm was measured using an ELISA reader. The endpoint titer was determined as two times the mean of the background OD value. Titers were calculated from the titration curve by interpolation unless the OD value was less than two times the mean of background at the starting dilution.

### Enzyme-Linked Immunospot (ELISPOT) Assays

To detect and quantify individual anti-OVA antibody-secreting B cells, splenocytes were analyzed using ELISPOT. Briefly, 96-well plates with PVDF membranes (Millipore, Burlington, MA) were coated with rOVA and incubated at 4°C for 18 hours. The plates were washed twice and blocked with RPMI medium supplemented with fetal bovine serum (10%) for 1 hour to prevent nonspecific binding. Splenocytes were seeded at a concentration of 1×10^5^ cells/well for 3 days. After incubation, the cells were removed from the plates by washing 3 times with 0.05% (w/v) Tween 20 in PBS (PBST). The biotinylated detection antibody was added to each well. The plates were then incubated at 37°C for 2 hours and washed 3 times with PBST. Then, avidin-horseradish peroxidase complex reagent was added for 45 min incubation at room temperature. After washing 3 times with PBST and then 3 times with PBS, 3-amine-9-ethyl carbazole (Sigma-Aldrich) staining solution was added to each well to develop the spots. The reaction was stopped by washing the plates with water. The spots were counted using an ELISPOT reader (Cellular Technology Ltd.).

The number of IFN-γ-producing cells in the spleen, superficial cervical lymph nodes and deep cervical lymph nodes was evaluated using mouse IFN-γ ELISPOT kits (BD Biosciences, San Jose, CA) according to the manufacturer’s instructions. The spots were determined as previously described (40).

### Quantification of Cytokine Release

The spleens were removed to create single-cell suspensions one week after the last immunization. Splenocytes were added into each well of a 24-well plate and were further stimulated with rOVA or BSA at 10 μg/mL. One well was set up for each stimulation. After culturing for 4 days, cell-free supernatants were harvested and stored at -80°C. The levels of IFN-γ, IL-5, IL-13 or IL-17A were measured using ELISA kits (eBioscience, San Diego, CA) according to the manufacturer’s instructions.

### Animal Challenge

Vaccine-immunized AG129 mice were intraperitoneally injected with 80 FFUs of Zika virus (PRVABC59) in 0.2 mL of PBS. Blood samples and vaginal wash samples were obtained 3 and 4 days post-Zika virus challenge, respectively.

### Focus-Forming Assays and Focus Reduction Neutralization Tests (FRNT)

Plasma and vaginal lavage collected from challenged mice at the indicated time points were diluted using 10-fold serial dilutions (starting at 1:10). Viral titers in the samples were determined by focus-forming assays as previously described (48). If the viral titers were less than 2.0 log10 FFU/mL (detection limit of the assay), a value of 1.0 was assigned for calculation purposes.

Neutralizing antibody titers were determined by FRNT as previously described (48). If the viral titers were less than 3.0 log2 (detection limit of the assay), a value of 2.0 was assigned for calculation purposes.

### Data Analysis

Values are expressed as the mean ± SEM. The Kruskal-Wallis test with Dunn’s multiple comparison was used to compare differences for more than two groups. Statistical analysis was performed using GraphPad Prism software version 5.02 (GraphPad Software, San Diego, CA). Differences with p < 0.05 were considered statistically significant.

## Results

### Intranasal Administration of rOVA-FLIPr Increases Antigen Deposition on the Nasal Mucosa and Efficiently Delivers OVA to DCs

**Figure 1:**
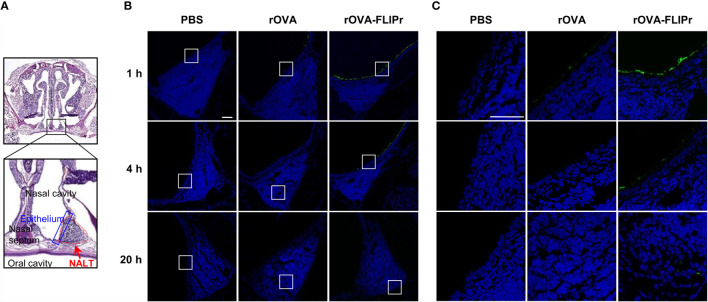
Ovalbumin fusion with FLIPr increases the deposition of ovalbumin on the nasal epithelium *via* intranasal administration. **(A)** Schematic position of NALT. Nasal cavity staining with hematoxylin and eosin. The red dotted line indicates the range of NALT, and the blue line indicates the epithelial layer that covers NALT. **(B)** PBS (left panel), rOVA (middle panel) or rOVA-FLIPr (right panel) was intranasally administered, and the nasal cavity was collected at the indicated time points. Antigen staining (green) in NALT was performed using an anti-His tag antibody following the Alexa Fluor^®^488-conjugated secondary antibody. Images were obtained from single optical sections acquired by means of confocal microscopy (40× total magnification, Scale bar = 100 µm). **(C)** The enlarged field of the white box from **(B)** (40× total magnification, Scale bar = 40 µm). Experiments were repeated twice with similar results.

To assess whether fusing antigen with FLIPr augments the delivery of antigen to the mucosal membrane and facilitates antigen capture by DCs in nasal lymphoid tissue (NALT), groups of C57BL/6 mice were intranasally administered 30 μg rOVA or rOVA-FLIPr. Mice administered PBS alone were used as controls. The nasal mucosal tissues (**Figure 1A**) were harvested 1, 4, and 20 hours after administration, and the presence of antigen was evaluated by immunofluorescence (**Figures 1B, C**). Background levels were observed in mice injected with PBS (**Figure 1B**, left panel). Some antigen-positive signals in the nasal mucosa were detected in rOVA-administered mice one hour after administration. Fluorescent intensities gradually waned and were barely detected 20 hours after administration (**Figure 1B**, middle panel). Compared to rOVA-injected mice, rOVA-FLIPr injection resulted in increased antigen deposition in the nasal mucosa (**Figure 1B**, right panel). Furthermore, antigens were distributed in the NALT and gradually increased in mice administered rOVA-FLIPr (**Figure 1C**). These results indicate that OVA fusion with FLIPr increases rOVA-FLIPr deposition in the nasal mucosa and promotes entry into the NALT.

**Figure 2:**
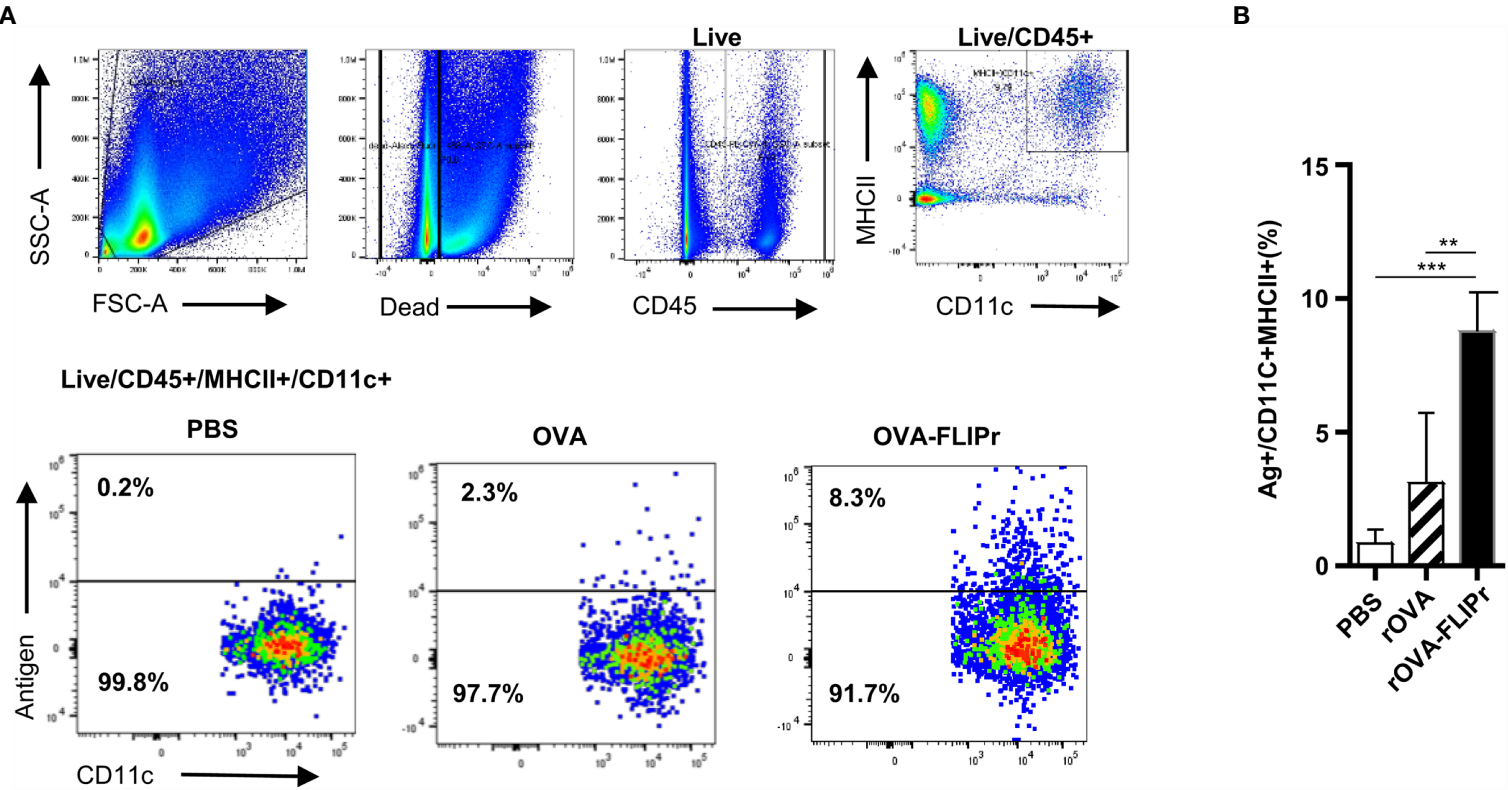
Ovalbumin fusion with FLIPr efficiently delivers to NALT dendritic cells *via* intranasal administration. **(A)** Gating strategy for the dendritic cell population in NALT. A group of C57BL/6 mice was intranasally administered 30 μg Alexa Fluor 647-labeled OVA or OVA-FLIPr. Mice administered PBS were used as control. Cells were harvested 2 hours after administration. A single-cell suspension of nasal cells (pooled from 3 mice) was obtained by mechanical disruption and collagenase digestion of nasal tissue. Dead cells were excluded from analysis by staining with a Live/Dead^®^ fixable dead cell stain dye. DCs from the NALT gated on live/CD45^+^/CD11c^+^MHCII^+^ cells. **(B)** The frequencies of antigen-labeled CD11c^+^MHC II^+^ cells. Cumulative data from three individual experiments are quantified here. The data are presented as the mean ± SEM (n = 3). Statistical significance was determined using the Kruskal-Wallis test with Dunn’s multiple comparison test. **p < 0.01; *** p < 0.001.

Since there was an increase in rOVA-FLIPr in the NALT, we next investigated whether rOVA-FLIPr is captured by DCs. Groups of mice were intranasally administered PBS, Alexa Fluor 647-conjugated rOVA or rOVA-FLIPr. The frequencies of fluorescence-containing CD11c^+^ MHC II^+^ cells in NALT (pooled from 3 mice) were analyzed by flow cytometry 2 hours after injection. The gating strategy and representative results are shown in **Figure 2A**. The frequency of fluorescence-containing CD11c^+^MHC II^+^ cells significantly increased in mice administered rOVA-FLIPr (**Figure 2B**). These results indicate that rOVA-FLIPr entering NALT can be captured by DCs. Furthermore, we examined the phenotype of DCs targeted by rOVA-FLIPr fusion protein. These DCs include CD103^+^CD11b^+^ and CD103^+^CD11b^-^ DC subsets (**Supplementary Figure S1**).

### Intranasal Vaccination With rOVA-FLIPr Elicits Long-Term Systemic and Mucosal Antibody Responses

**Figure 3:**
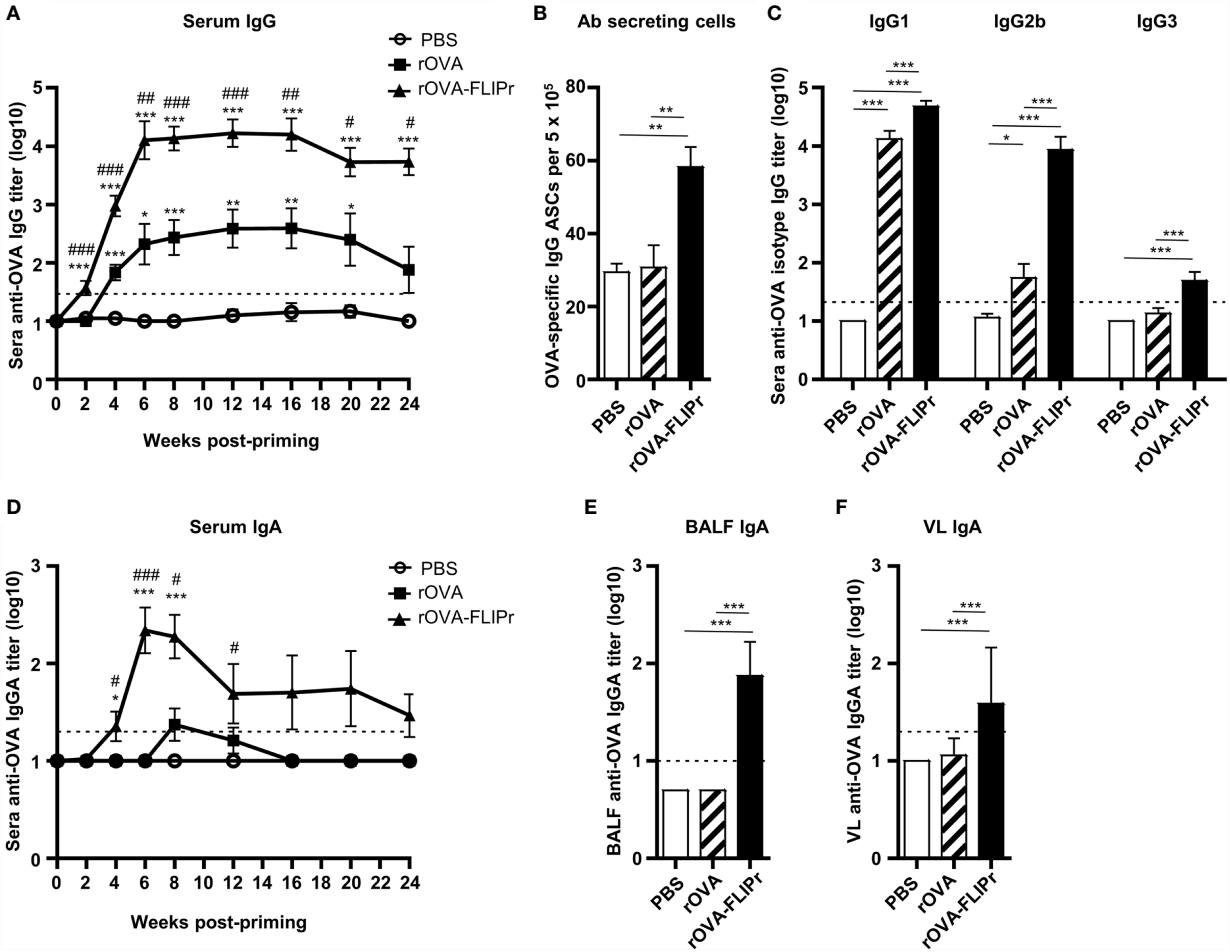
Antibody responses induced by intranasal administration of rOVA-FLIPr. Groups of C57BL/6 mice (n=6/group) were intranasally immunized three times with 30 μg of OVA or OVA-FLIPr in PBS at 2-week intervals. Mice immunized with PBS alone (without antigens) served as controls. Sera were collected at the indicated time points. **(A, D)** OVA-specific IgG and IgA was assessed by ELISA. Data represent the mean ± SE of the mean. Statistical significance was determined using the Kruskal-Wallis test with Dunn’s multiple comparison test. *p < 0.05; **p < 0.01; ***p < 0.001 v.s. PBS. #p < 0.05; ##p < 0.01; ###p < 0.001 v.s. rOVA. **(B)** OVA-specific antibody-secreting cells in splenocytes were evaluated using ELISPOT at 24 weeks after the first vaccination. **(C)** Reactivity of subtype IgG1, IgG2b, and IgG3 antibodies specific to OVA. **(E, F)** Reactivity of OVA-specific IgA antibody titers in BALF and VL was assessed by ELISA. The BALF and VL were collected 6 weeks after the first vaccination. Data represent the mean ± SE of the mean. The detection limit is indicated by the dotted line on the y-axis, which indicates the initial dilution factor of the sample. Statistical significance was determined using the Kruskal-Wallis test with Dunn’s multiple comparison test. *p < 0.05; **p < 0.01; ***p < 0.001. The results are representative of three independent experiments.

To determine the immune response induced by rOVA-FLIPr, groups of C57BL/6 mice received three immunizations with PBS, rOVA, or rOVA-FLIPr (30 μg per dose) with a two-week interval between immunizations. Serum samples were collected from the immunized mice at different time points as indicated. As shown in **Figure 3A**, level of OVA-specific IgG in sera were quickly increased in mice that received one dose of rOVA-FLIPr (2 weeks post priming). After booster vaccination, anti-OVA IgG antibody titers were further increased and reached a peak level at 6 weeks post priming. Antibody responses were sustained over 24 weeks post priming. Anti-OVA IgG antibody titers in mice immunized with rOVA-FLIPr were significantly higher than those in mice immunized with rOVA at all time points examined. Consistent with anti-OVA IgG antibody titers in sera, antibody-secreting cells in splenocytes were still detected 24 weeks post priming (**Figure 3B**). Next, we evaluated the serum IgG subtype in the vaccinated mice. Both rOVA-FLIPr- and rOVA-immunized mice elicited OVA-specific IgG1 antibody responses (**Figure 3C**). Interestingly, only mice immunized with rOVA-FLIPr exhibited IgG2b and IgG3 antibody responses. These results suggest that intranasal vaccination with rOVA-FLIPr elicits long-term systemic IgG antibody responses and diverse IgG subtypes.

Furthermore, we evaluated IgA antibody responses. Levels of OVA-specific IgA were markedly increased in the sera of rOVA-FLIPr-immunized mice 4 weeks post priming and reached its peak level at 6 weeks post priming. In contrast, few or no OVA-specific IgA antibodies were detected in the sera of rOVA- or PBS-immunized mice (**Figure 3D**). Since mice immunized with rOVA-FLIPr induced OVA-specific IgA antibodies in the sera, we further examined IgA levels in bronchoalveolar lavage fluid (BALF) and vaginal lavage (VL). In line with sera IgA, OVA-specific IgA antibodies were present in the BALF and VL obtained from mice immunized with rOVA-FLIPr. There were no detectable OVA-specific IgA antibodies in the BALF or VL obtained from mice immunized with rOVA or PBS (**Figures 3E, F**). These results suggest that intranasal vaccination with rOVA-FLIPr induces mucosal antibody responses.

### Intranasal Vaccination With rOVA-FLIPr Induces Broad-Spectrum Antigen-Specific T Cell Responses

**Figure 4:**
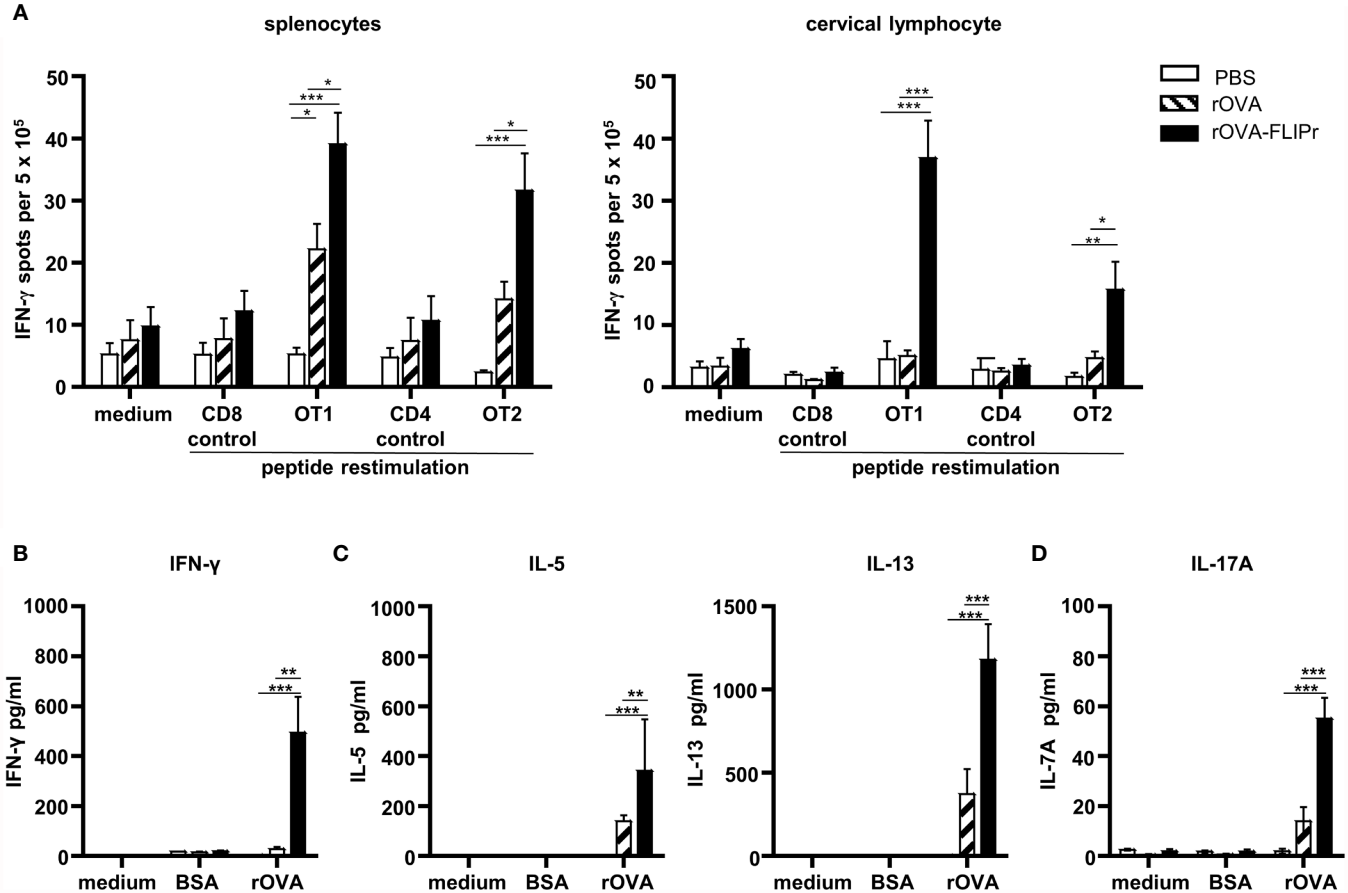
T cell responses and cytokine production profiles induced by intranasal administration of rOVA-FLIPr. Groups of C57BL/6 mice (n=5/group) were immunized three times intranasally with 30 μg of OVA or OVA-FLIPr in PBS at 2-week intervals. Mice immunized with PBS alone (without antigens) served as controls. **(A)** Splenocytes and cervical lymphocytes were harvested one week after the last immunization. Cells were cultured and stimulated with OT1, OT2, or control peptides for 3 days in an anti-INF-γ-coated 96-well ELISPOT plate. IFN-γ responses were measured using ELISPOT assay and are expressed as spot-forming units per 5×10^5^ cells. **(B–D)** Splenocytes were cultured and stimulated with rOVA for 4 days. Stimulation with BSA or medium alone served as controls. The supernatants were collected to evaluate levels of **(B)** IFN-γ, **(C)** IL-5, IL-13 and **(D)** IL-17A by ELISA. Data represent the mean ± SE of the mean. The results shown are from one of two representative experiments. Statistical significance was determined using the Kruskal-Wallis test with Dunn’s multiple comparison test. *p < 0.05; **p < 0.01; *** p < 0.001.

In addition to antibody production, we further evaluated cellular immune responses potentially induced by intranasal vaccination of C57BL/6 mice with rOVA-FLIPr or rOVA. In parallel, mice immunized with PBS alone served as negative controls. The frequencies of IFN-γ-secreting cells in the spleens and cervical lymph nodes (obtained from the superficial cervical lymph nodes and deep cervical lymph nodes) were examined one week after the last immunization. For all of the splenocytes and cervical lymphocytes, the frequencies of IFN-γ-secreting cells were at background levels when there was no stimulation (medium alone) or stimulation with control peptides. Remarkably, the splenocytes and cervical lymphocytes obtained from mice immunized with rOVA-FLIPr exhibited in higher frequencies of IFN-γ-secreting cells than mice immunized with rOVA or PBS after stimulation with OT-1 (a CD8 epitope) or OT-2 (a CD4 epitope) peptides (**Figure 4A**). These results indicate that intranasal vaccination with rOVA-FLIPr induces OVA-specific CD4^+^ and CD8^+^ T cell responses.

Next, the levels of Th1-associated cytokines (IFN-γ), Th2-associated cytokines (IL-5 and IL-13) and Th17-associated cytokines (IL-17A) produced from splenocytes of vaccine-immunized mice were evaluated. Supernatants obtained from all of the splenocytes produced few or background levels of IFN-γ, IL-5, IL13, and IL-17A without stimulation (medium alone) or when stimulated with bovine serum albumin (BSA). Notably, supernatants obtained from mice immunized with rOVA-FLIPr contained higher levels of IFN-γ, IL-5, IL13, and IL-17A than mice immunized with rOVA or PBS after stimulation with rOVA (**Figures 4B–D**). These results suggest that intranasal vaccination with rOVA-FLIPr triggers CD4^+^ and CD8^+^ T cell responses and induces a broad-spectrum cytokine secretion profile.

### Intranasal Vaccination With Recombinant Zika Virus Envelope Protein Domain III-FLIPr (rZEIII-FLIPr) Elicits Immune Responses With Protective Effects

**Figure 5:**
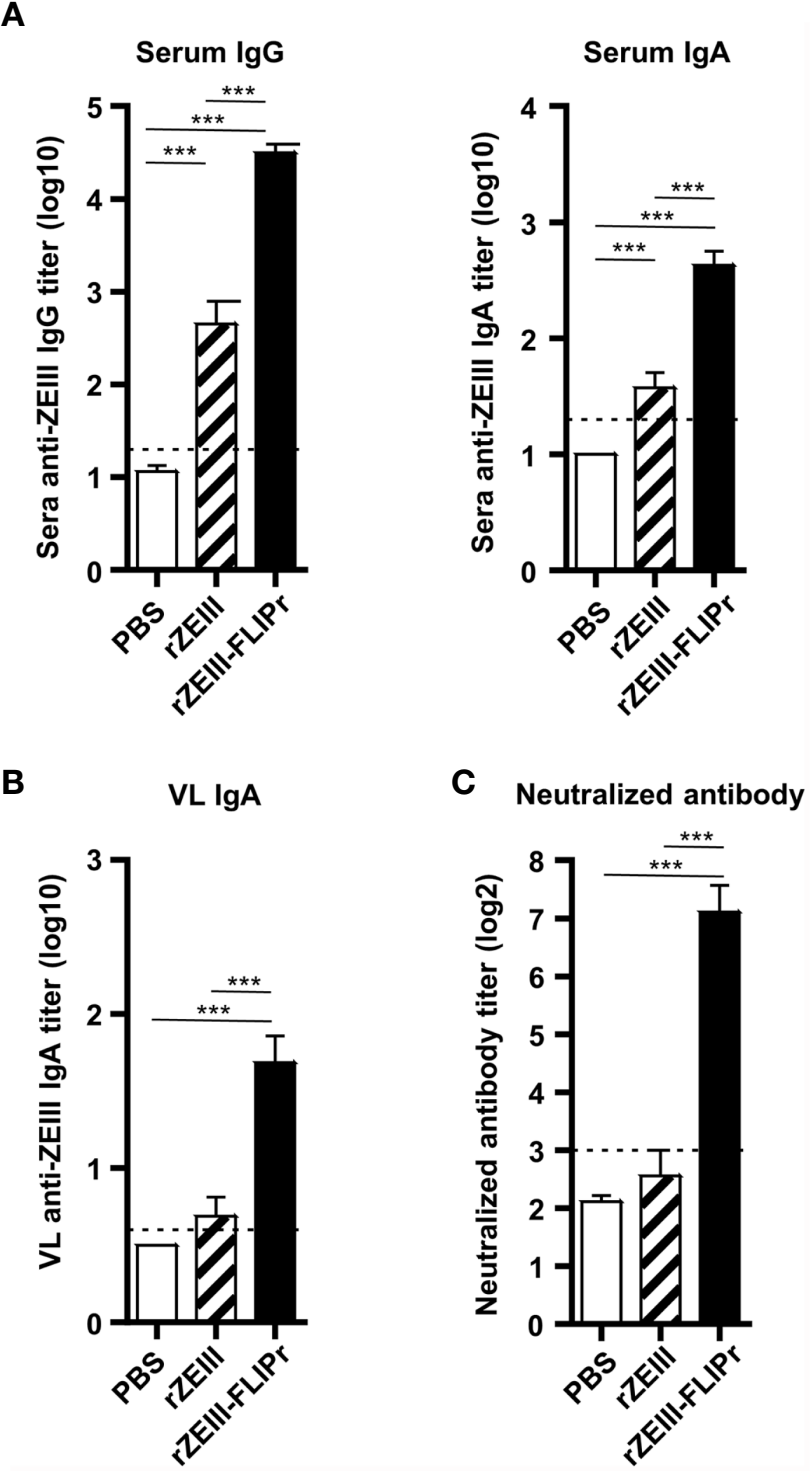
Antibody responses induced by intranasal administration of rZEIII-FLIPr. Immunodeficient AG129 mice (n=9/group) were vaccinated three times with PBS, rZEIII, or rZEIII-FLIPr (30 μg per dose) *via* the intranasal route at two-week intervals. Serum and VL samples were collected from vaccinated mice 6 weeks after the first vaccination. **(A)** The titers of anti-rZEIII IgG and IgA antibodies in the serum were determined by ELISA. **(B)** The titers of anti-rZEIII IgA antibodies in VL were determined by ELISA. **(C)** The Zika virus-neutralizing capacity of the serum samples was determined by FRNT. The neutralizing antibody titer was defined as the reciprocal of the highest dilution that resulted in a 50% reduction in FFUs compared to the FFUs of control samples containing the virus alone. Data represent the mean ± SE of a total of 9 mice per group, which were pooled from 2 independent experiments. The detection limit is indicated by the dotted line on the y-axis, which indicates the initial dilution factor of the sample. Statistical significance was determined using the Kruskal-Wallis test with Dunn’s multiple comparison test. ***p < 0.001.

In view of the strong capability of rOVA-FLIPr to induce broad immune responses, we next produced rZEIII-FLIPr and evaluated the potential of rZEIII-FLIPr as a vaccine candidate against Zika virus. Groups of immunodeficient mice (AG129) or immunocompetent mice (C57BL/6) received three intranasal immunizations with PBS, rZEIII or rZEIII-FLIPr with a two-week interval between immunizations. The ZEIII-specific IgG and IgA titers in sera 6 weeks after priming indicated that mice immunized with rZEIII-FLIPr induced superior systemic antibody responses to mice immunized with rZEIII (**Figure 5A** and **Supplementary Figure S2A**). In addition, we examined IgA titers in VL. We found that only rZEIII-FLIPr-immunized mice were able to notably induce anti-ZEIII IgA antibody responses in the VL (**Figure 5B** and **Supplementary Figure S2B**). These results are consistent with the rOVA-FLIPr studies.

Next, we evaluated the neutralizing capacity in the sera elicited by vaccination. As shown in **Figure 5C** and **Supplementary Figure S2C**, PBS- or rZEIII-immunized mice were unable to induce neutralizing antibody responses. In contrast, significant neutralizing antibody responses were observed in the group of rZEIII-FLIPr-immunized mice. These results suggest that mice intranasally immunized with rZEIII-FLIPr in an exogenous adjuvant-free formulation develop neutralizing antibody responses.

**Figure 6:**
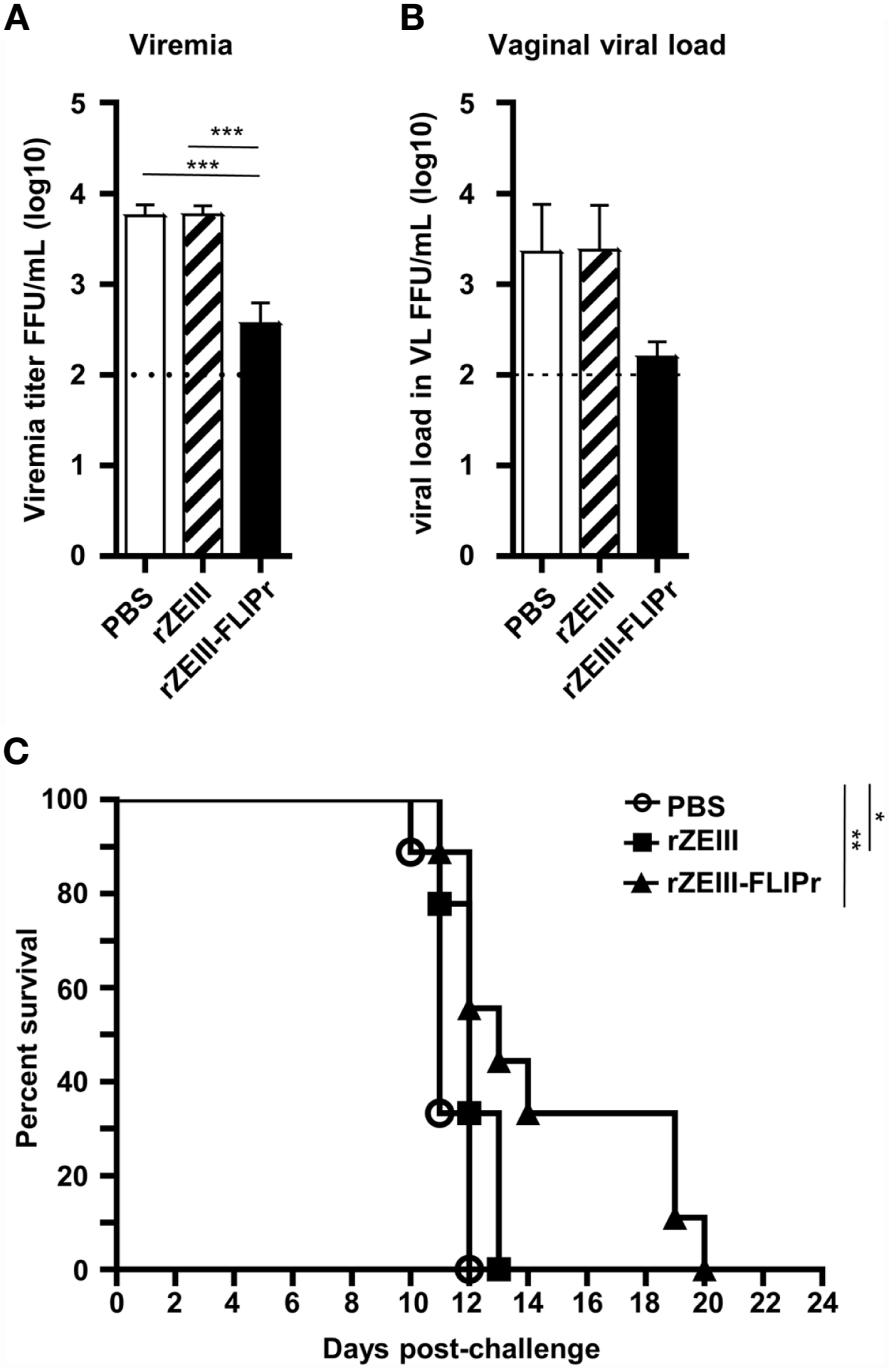
Protective effects induced by intranasal administration of rZEIII-FLIPr. Groups of immunodeficient AG129 mice (n = 9/group) were intranasally administered PBS, rZEIII, or rZEIII-FLIPr (30 μg per dose) 3 times at two-week intervals. All animals were intraperitoneally injected with 80 FFUs of Zika virus (PRVABC59) in 0.2 mL of PBS at 6 weeks after the first administration. **(A)** Plasma and **(B)** VL samples were collected 3 and 4 days after virus challenge, respectively. The viral titers were evaluated by focus-forming assays. The viral titers were logarithmically transformed before statistical analyses. Data represent the mean ± SE of the mean. Statistical significance was determined using the Kruskal-Wallis test with Dunn’s multiple comparison test. ***p<0.001. **(C)** The overall survival of the mice is shown. Survival analysis was calculated by Log-Rank test. *p < 0.05. **p < 0.01.

To investigate the protective potential induced by rZEIII-FLIPr against Zika virus infection, AG129 mice were immunized with PBS, rZEIII, or rZEIII-FLIPr and then challenged with Zika virus 6 weeks after priming. The viral load in the sera and vaginal wash samples from rZEIII-FLIPr-immunized mice was lower than that in rZEIII- or PBS-immunized mice (**Figures 6A, B**). Importantly, the rZEIII-FLIPr-immunized mice exhibited prolonged survival times compared to the PBS- or rZEIII-immunized mice (**Figure 6C**). These results suggest that intranasal vaccination with rZEIII-FLIPr induces protective immune responses to decrease viral load and prolong survival times.

## Discussion

In this study, we applied an antigen-FLIPr fusion protein alone to generate mucosal immune responses. Our results demonstrated that FLIPr is an effective vector that elicits mucosal and systemic immune responses *via* intranasal administration.

Although mucosal vaccination is considered an attractive route for vaccine delivery, one of the primary challenge of intranasal vaccination is weak immune responses resulting from inefficient antigen transportation across mucosal barriers into the immune inductive site (15), such as NALT in rodents. NALT is remarkably infiltrated by lymphocytes, including DCs, macrophages, B cells and T cells, which form an organized structure under the epithelial layer that collaborates to induce an immune response (50, 51). Considering the safety and efficacy of mucosal vaccines, the method of strategically delivering antigens to DCs without the use of adjuvants is a suitable choice. After intranasal administration of rOVA-FLIPr alone, a substantial amount of rOVA-FLIPr adheres to the nasal mucosa and then further penetrates NALT (**Figure 1**) and targets DCs (**Figure 2**). These results are conducive to the subsequent induction of antigen-specific immune responses.

NALT contains all the lymphocytes needed to induce and regulate mucosal immune responses after antigens delivered to the nasal cavity (52). In addition, NALT is a mucosal inductive site for humoral and cellular immunities with local and systemic responses (53, 54). Among the CD11c^+^ MHCII^+^ nasal cells, five distinct populations of antigen presenting cells have been identified (51). CD103^+^ CD11b^-^ cells and CD103^+^ CD11b^+^ cells are predominantly associated with DC characterization. CD103^-^ CD11b^+^ cells, including 3 subsets, are associated with more macrophage characterization. CD103^+^ DCs play an important role in IgA production (55, 56) and intestinal mucosal immune responses (57). In this study, rOVA-FLIPr fusion protein is superior to rOVA in targeting to DCs, including CD103^+^ CD11b^-^ and CD103^+^ CD11b^+^ DCs (**Supplementary Figure S1**). Therefore, antigen loaded DCs migrate to NALT then trigger robust immune responses. This notion is in line with the antigen-specific IgA and IgG production in antigen-FLIPr vaccinated mice (**Figures 3**, **5**), long-term antibody-secreting cells (**Figure 3B**), CD4^+^ and CD8^+^ T cell responses (**Figure 4**) and a broad-spectrum cytokine secretion profile (**Figures 4B–D**).

Indeed, immunization of rOVA-FLIPr *via* intranasal administration induced long-lasting anti-OVA IgG and IgA antibody responses (**Figure 3**). Many studies have shown that it is necessary to rely on mucosal vaccination to produce a strong IgA response (21, 58). IgA exists in the circulatory system and the mucosal surface; the former exists in the form of monomer or polymer (pIgA), while the latter is in the form of a dimer and can be secreted to the mucosal surface (sIgA). sIgA protects the host by binding to the surface of luminal pathogens and toxins, preventing them from attaching to epithelial cells (59, 60). We show that intranasal administration of rOVA-FLIPr alone, but not intranasal administration of rOVA alone, successfully induced IgA in the circulatory system or mucosal compartments, including bronchoalveolar and vaginal compartments (**Figures 3D** **–F**). In addition to the marked IgA-inducing ability of rOVA-FLIPr, we also observed that intranasal administration of rOVA-FLIPr alone induced not only IgG1 but also IgG2b and IgG3 antibody responses. These results suggest that FLIPr is a potent vector for intranasal administration to induce mucosal immune responses as well as diverse antibody responses. Here, we employed ZEIII as an immunogen, which has been reported to induce neutralizing antibodies against the Zika virus (61, 62). Consistent with rOVA-FLIPr, intranasal administration of rZEIII-FLIPr alone was superior to rZEIII in inducing not only serum IgG and IgA (**Figure 5A**) but also sIgA in the vagina (**Figure 5B**). Notably, sera obtained from mice immunized with rZEIII-FLIPr, but not rZEIII, had the capacity to block Zika virus infection (**Figure 5C**). These results indicate that superior immunogenicity of antigen by fusing antigen with FLIPr is not antigen dependent.

Mice are not the natural host of Zika virus. However, both immunocompetent (C57BL/6 or BALB/c) (63) and immunodeficient (A129 or AG129) mouse infection models are used for Zika virus studies (64, 65). The infection route for immunocompetent mice is *via* intracerebral injection. For immunodeficient mice, infection route is *via* intraperitoneal injection, foot pad injection, or intravenous injection. Mouse strain, infection route, infectious dose, and virus strain may result different outcomes. In this proof-of-concept study, we infect immunodeficient AG129 mice *via* intraperitoneal injection to evaluate the vaccine efficacy. In line with antibody responses, intranasal administration of rZEIII-FLIPr alone effectively reduced the viral load in the blood (**Figure 6A**) and vagina (**Figure 6B**) after Zika virus infection. Furthermore, the survival times of these mice were prolonged (**Figure 6C**). These results suggest that rZEIII-FLIPr is a potential vaccine candidate against the Zika virus.

In addition to the humoral immune response, the cellular immune response is another mainstay of adaptive immunity. In the present study, we demonstrate that direct intranasal administration of rOVA-FLIPr activates both OVA-specific CD4 and CD8 T cell responses. These activated T cells were detected in the spleen and cervical lymph nodes (**Figure 4A**). Different T cell types secrete various cytokines and play different roles in the immune system. We demonstrated that rOVA-FLIPr is superior to rOVA in stimulating cytokine production for all cytokines we examined (**Figure 4B**). These cytokines include Th1-type cytokines (IFN-γ), Th2-type cytokines (IL-5 and IL-13), and Th17-type cytokines (IL-17A). These results support that the antigen-FLIPr fusion protein alone is able to stimulate broad-spectrum T cell responses.

In conclusion, our results show that intranasal administration of antigen-FLIPr fusion protein alone does not require exogenous adjuvant or complicated formulation and efficiently targets antigen to DCs, triggering mucosal and systemic antibody responses as well as broad-spectrum T cell responses. We further applied Zika virus vaccine development and demonstrated that rZEIII-FLIPr is a potent vaccine candidate that elicits protective immune responses against the Zika virus. This implies that antigen fusion with FLIPr is an effective strategy for vaccine development against infectious diseases and cancer immunotherapy.

## Data Availability Statement

The original contributions presented in the study are included in the article/**Supplementary Material**. Further inquiries can be directed to the corresponding author.

## Ethics Statement

The animal study was reviewed and approved by the Animal Committee of the National Health Research Institutes.

## Author Contributions

M-SH and H-WC contributed to the study concept and design. M-SH and H-WC wrote the initial draft of the manuscript. M-SH, C-WH, and L-LT performed experiments and analyzed data. KC, L-LY, C-CW, M-YC, and C-YC prepared recombinant proteins and virus. S-JL, C-LL, and H-WC supervised the study. All authors contributed to the article and approved the submitted version.

## Funding

This work was supported by grants from the Ministry of Science and Technology, Taiwan (MOST 106-2321-B-400-009, MOST 107-2321-B-400-006, and MOST 108-2321-B-400-012) and National Health Research Institutes, Taiwan (IV-106-PP-18 and IV-107-PP-18).

## Conflict of Interest

H-WC and S-JL are named on patent applications relating to a method for the enhancement of immune responses using an antigen fusion protein containing an antigen and an antagonist of an Fc gamma receptor.

The remaining authors declare that the research was conducted in the absence of any commercial or financial relationships that could be construed as a potential conflict of interest.

## Publisher’s Note

All claims expressed in this article are solely those of the authors and do not necessarily represent those of their affiliated organizations, or those of the publisher, the editors and the reviewers. Any product that may be evaluated in this article, or claim that may be made by its manufacturer, is not guaranteed or endorsed by the publisher.
