# Supplementary material for: Intranasal Vaccination With Recombinant Antigen-FLIPr Fusion Protein Alone Induces Long-Lasting Systemic Antibody Responses and Broad T Cell Responses
Source: Front Immunol. 2021 Oct 11;12:751883. doi: 10.3389/fimmu.2021.751883 (PMC8543008; doi:10.3389/fimmu.2021.751883)
Supplement: Supplementary file 1 [file DataSheet_1.pdf]

Supplementary Material

Intranasal vaccination with recombinant antigen-FLIPr fusion protein alone induces long-lasting systemic antibody responses and broad T cell responses

Ming-Shu Hsieh[1], Chia-Wei Hsu[1], Ling-Ling Tu[1], Kit Man Chai[1], Li-Lu Yu[1], Chiao-Chieh Wu[1], Mei-Yu Chen[1], Chen-Yi Chiang[1], Shih-Jen Liu[1, 2, 3], Ching-Len Liao[1], Hsin-Wei Chen[1, 2, 3*]

[1]National Institute of Infectious Diseases and Vaccinology, National Health Research Institutes, Miaoli, 35053, Taiwan.
[2]Graduate Institute of Biomedical Sciences, China Medical University, Taichung, 406040, Taiwan.
[3]Graduate Institute of Medicine, Kaohsiung Medical University, Kaohsiung, 307378, Taiwan.

*Correspondence:
Hsin-Wei Chen (Email: chenhw@nhri.org.tw)

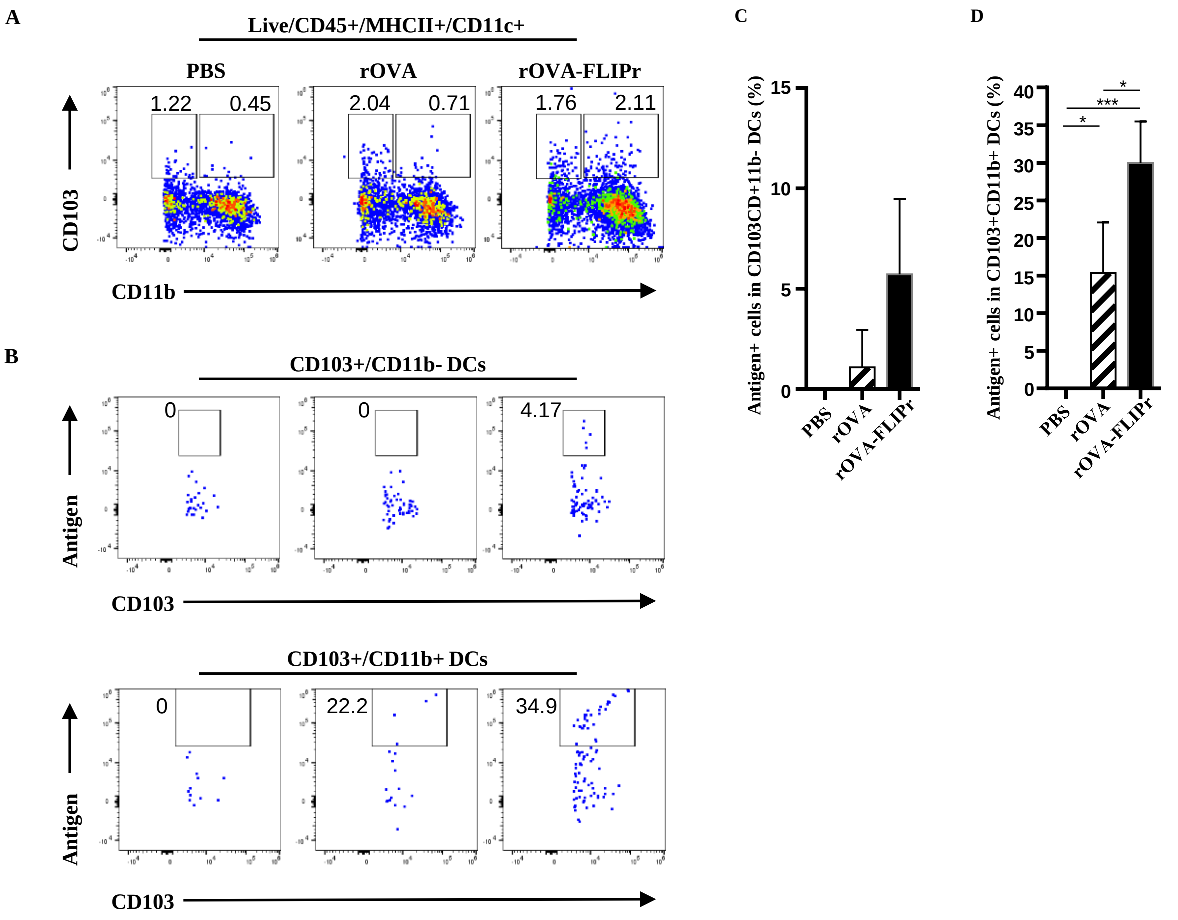

Supplementary Figure S1. Ovalbumin fusion with FLIPr efficiently delivers to NALT CD103^{+} dendritic cells via intranasal administration. (A) NALT DCs gated on live/CD45+/CD11c+MHCII+ cells are separated by the expression of CD103 and CD11b. **(B)** The representative figure of antigen-labeled CD103^{+}CD11b^{-} or CD103^{+}CD11b^{+} DCs. The box representative the antigen positive cells. **(C)**The frequencies of antigen-labeled CD103^{+}CD11b^{-} DCs. **(D)** The frequencies of antigen-labeled CD11c^{+}MHCII^{+}CD103^{+}CD11b^{-} DCs. Cumulative data from three individual experiments are quantified here. The data are presented as the mean ± SEM (n=3).

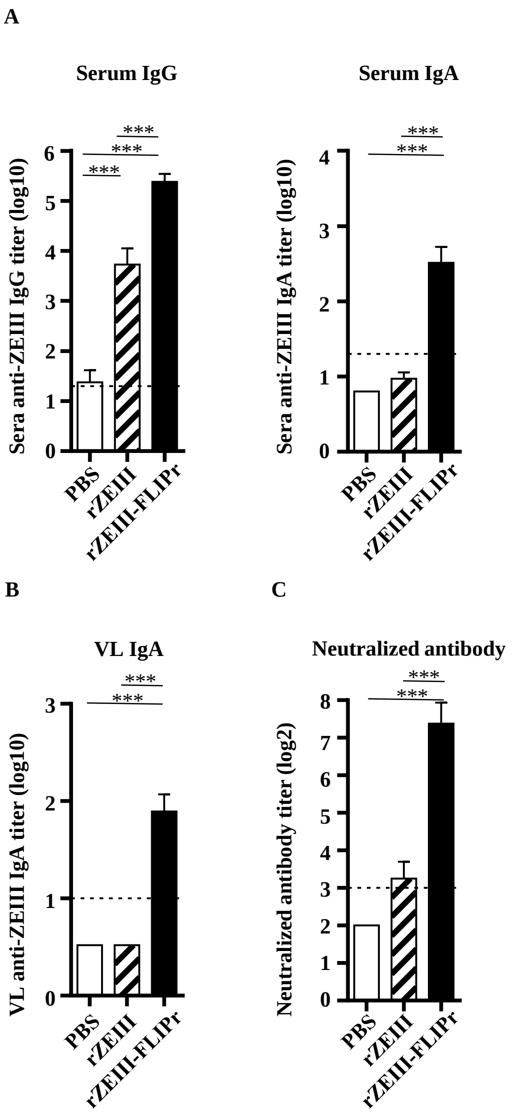

Supplementary Figure S2. Antibody responses induced by intranasal administration of rZEIII-FLIPr in C57BL/6 mice. Immunocompetent C57BL/6 mice (n=8/group) were vaccinated three times with PBS, rZEIII, or rZEIII-FLIPr (30 µg per dose) via the intranasal route at two-week intervals. Serum and VL samples were collected from vaccinated mice 6 weeks after the first vaccination. **(A)** The titers of anti-rZEIII IgG and IgA antibodies in the serum were determined by ELISA. **(B)** The titers of anti-rZEIII IgA antibodies in VL were determined by ELISA. **(C)** The Zika virus-neutralizing capacity of the serum samples was determined by FRNT. The neutralizing antibody titer was defined as the reciprocal of the highest dilution that resulted in a 50% reduction in FFUs compared to the FFUs of control samples containing the virus alone. Data represent the mean ± SE of a total of 9 mice per group, which were pooled from 2 independent experiments. The detection limit is indicated by the dotted line on the y-axis, which indicates the initial dilution factor of the sample. Statistical significance was determined using the Kruskal-Wallis test with Dunn's multiple comparison test. ***$p < 0.001$.
